# Evaluation of the amount of residual lipid emulsion in chambers of flushed totally implantable venous access devices using fluorescence imaging

**DOI:** 10.1038/s41430-019-0443-8

**Published:** 2019-06-04

**Authors:** Naoya Okamura, Takae Yamato, Ippei Yamaoka

**Affiliations:** 1 Information Center for Infusion Therapy and Product, Otsuka Pharmaceutical Factory, Inc., 115 Kuguhara, Tateiwa, Muya-cho, Naruto, Tokushima 772-8601 Japan; 2 Medical Foods Research Institute, Otsuka Pharmaceutical Factory, Inc., 115 Kuguhara, Tateiwa, Muya-cho, Naruto, Tokushima 772-8601 Japan

**Keywords:** Risk factors, Imaging

## Abstract

The aim of the present study was to use a quantitative fluorescence imaging technique to evaluate the invisible amount of residual lipid emulsion in port chambers flushed with various fundamental protocols. Chambers were filled with lipid emulsion containing indocyanine green and then flushed with 5–70 mL of normal saline. Chambers were flushed at various speeds (15–60 mL/min), with a time interval of 1 or 3 s between boluses, and with varying directions of flow. The slower the flushing speed, the more lipid emulsion that remained. Pulsatile flushing with either time interval did not decrease the residual amounts, and the areas well-cleansed after flushing were oriented to the bevel-opening direction. These findings suggest that to reduce the residual amount of lipid emulsion poured in a chamber, fast and furious flushing under continuous as opposed to pulsatile flushing is of paramount importance.

## Introduction

Occlusion, a representative complication associated with totally implantable venous access devices (TIVADs), can lead to failure in successive infusions. Occlusions are occasionally caused by lipid aggregates, thrombus formation, or the precipitation of drugs or calcium salts [1, 2]. Moreover, residual lipid emulsion in port chambers is a significant risk factor for catheter-related bloodstream infections because lipids have been identified as a microorganism growth factor [3].

Therefore after lipid infusion, the patency of the device necessitates appropriate flushing with saline. To perform appropriate flushing, the behavior of negligible residues in the device needs to be clarified. Hence, we evaluated the effectiveness of different flushing techniques for lipid emulsion in TIVAD port chambers using indocyanine green (ICG) fluorescence imaging.

## Materials and methods

### Preparation for filling port chambers with lipid emulsion

The lipid emulsion used in the present study consisted of Intralipos® injection 20% (soybean oil 200 mg/mL; Otsuka Pharmaceutical Factory, Inc., Tokushima, Japan) and ICG (25 mg in 100 mL of Intralipos® injection 20%, Diagnogreen® for injection 25 mg; Daiichi Sankyo Company, Ltd., Tokyo, Japan).

A 22-gauge Huber-point needle (Huber Plus® Non-coring Needle; C. R. Bard, Inc., Murray Hill, NJ, USA) was inserted into the central venous (CV) port (X-Port isp™; C. R. Bard, Inc.) and the other end was connected to a closed connector (Bionector S; Vygon Japan Corp., Osaka, Japan). Finally, the prepared lipid emulsion was filled using a common infusion pump.

### Measurement of lipid emulsion using fluorescence imaging

The residual amounts of lipid emulsion in the chambers were calculated using the absolute calibration method with reference to the standard curves for fluorescence intensities and the amounts of lipid emulsion quantified by turbidity.

### Evaluation of factors affecting the flushing efficacy of lipid emulsion in the chamber

Next, the closed connector was connected to a mechanical syringe pump (YMC Co., Ltd., Kyoto, Japan) and then flushed with 5, 10, 20, 40, or 70 mL saline under the following experiments:

1. Effect of flushing speed: the chamber was continuously flushed at a speed of 15, 30, 40, 50, or 60 mL/min;
2. Effect of pulsatile flushing: 1 mL of normal saline flushing at a speed of 30 or 60 mL/min was followed by 1 or 3 s of holding;
3. Effect of the hole orientation of the Huber needle: the needle was fixed in a central position and then placed so that the angle formed by the needle hole and the exit catheter was 0°, 90°, or 180°.

These experiments were repeated three times under the same experimental conditions. The residual lipid emulsion in the chamber was calculated by monitoring the ICG using the IVIS Spectrum live imaging system (Perkin Elmer, Boston, MA, USA) [4].

### Statistical methods

All values are presented as means ± standard deviation. Differences between the treatments were analyzed using repeated-measures analysis of variance (ANOVA), followed by two-tailed Tukey’s test when appropriate. The level of statistical significance was set at *p* < 0.05. Ekuseru–Toukei 2015 statistical software (SSRI Co., Ltd., Tokyo, Japan) was used for all statistical analyses.

## Results

**Fig. 1:**
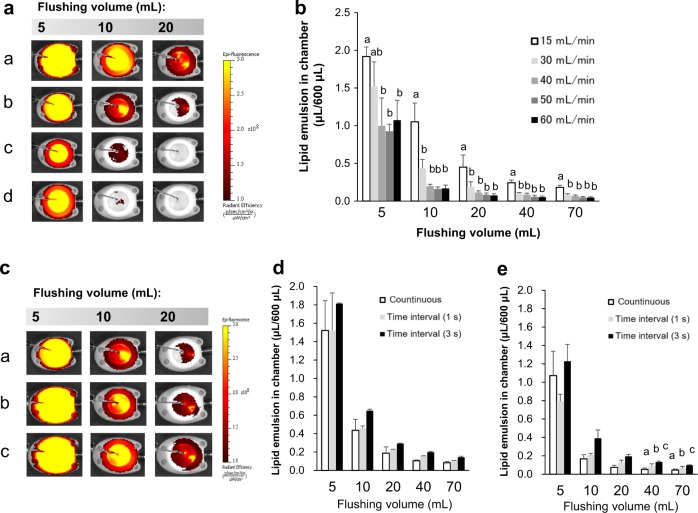
Representative fluorescence images of residual lipid emulsion after continuous flushing at 15 (a), 30 (b), 40 (c), or 60 (d) mL/min until reaching flushing volume (in **a**), and with or without (a) a time interval of 1 s (b) or 3 s (c) between boluses at 30 mL/min (in **c**). The residual amounts at various speeds (15–60 mL/min) until reaching flushing volume (in **b**) and between continuous and pulse intermittent flushing at 30 mL/min (in **d**) and 60 mL/min (in **e**). Values are expressed as mean ± standard deviation (*n* = 3). Different small letters indicate significant differences between groups (*p* < 0.05)

Speeds at <30 mL/min left behind ICG fluorescence; by contrast, speeds at >40 mL/min did not (Fig. 1a). As shown in Fig. 1b, the higher the flushing speed, the lower the residual amounts of lipid emulsion (*p* < 0.05; repeated-measures ANOVA). However, the residual amounts of lipid emulsion did not significantly differ when the flushing volume was increased to over 10 mL in the case of >30 mL/min.

At a speed of 30 mL/min, the residual amounts after pulsatile flushing with a time interval of 1 or 3 s between boluses were not lower than those after continuous flushing (Fig. 1d). The pulsatile flushing left higher residual amounts with higher hold times at 60 mL/min (3 s > 1 s > 0 s; *p* < 0.05; repeated-measures ANOVA).

**Fig. 2:**
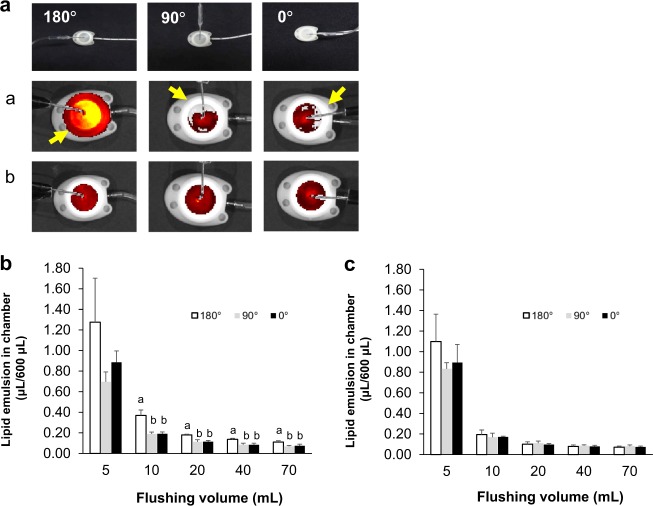
Representative bright-field and fluorescence images of chambers continuously flushed with 20 mL at 30 (a) or 60 (b) mL/min at various insertion angles (in **a**). The residual amounts among various insertion angles after continuous flushing at 30 (in **b**) or 60 (in **c**) mL/min in each volume with 5–70 mL. Values are expressed as mean ± standard deviation (*n* = 3). Different small letters indicate significant differences between groups (*p* < 0.05)

Figure 2a shows that the well-cleansed area corresponded to the hole orientation at a flushing speed of 30 mL/min, but when the flushing speed was 60 mL/min, the entire area was uniformly cleansed. When the hole orientation was opposite to the outlet catheter, more lipid emulsion remained compared with the other orientations examined (Fig. 2b).

## Discussion

The present findings indicate that the faster the flushing speed and/or the higher the volume of normal saline, the lower the amount of residual lipid emulsion remaining in the chamber. If the flushing speed is slow, it cannot homogenously stir the lipid emulsion, resulting in a larger pool of lipid emulsion inside the chamber. By contrast, a comparably faster flushing speed could produce a turbulent flow inside the chamber, resulting in the adequate stirring of the lipid emulsion and a smaller pool of lipid emulsion inside the chamber. Flushing with 10–20 mL of normal saline using a CV catheter is recommended in clinical practice [1]. The results of the present study support this recommendation, but the flushing speed should exceed 40 mL/min.

Pulsatile flushing did not decrease the residual amounts of lipid emulsion compared with the continuous controls, and a longer time interval between the boluses reduced the cleansing performance in the case of higher flushing speeds. The interruption of turbulent flow by interval handling made it difficult to maintain turbulent flow in chambers, which resulted in a reduced cleansing effect. This result is supported by idea that the turbulent flow readily disappears if the flow continues to be blocked [5]. There may be differences in the cleansing efficacy between chambers with a wide space and catheters with a narrow lumen, as the effectiveness of pulsatile compared with continuous flushing has been demonstrated [6, 7].

The present findings may indicate that the direction of Huber-point needle bevel produces an area where lipid emulsion easily remains at slower flushing speeds. However, turbulent flow occurs in the chamber if the speed of the flushing flow is high; therefore, the lipid emulsion in the chamber might be cleansed evenly, regardless of the direction of the Huber-point needle bevel.

In conclusion, to reduce the residual amount of lipid emulsion in a chamber, fast and energetic continuous flushing, as opposed to pulsatile flushing, is of paramount importance.
